# Ventilatory response to exercise in adolescents with cystic fibrosis and mild-to-moderate airway obstruction

**DOI:** 10.1186/2193-1801-3-696

**Published:** 2014-11-27

**Authors:** Bart C Bongers, Maarten S Werkman, Tim Takken, Erik H J Hulzebos

**Affiliations:** Department of Epidemiology, School for Public Health and Primary Care (CAPHRI), Maastricht University, Maastricht, The Netherlands; Child Development & Exercise Center, Wilhelmina Children’s Hospital, University Medical Center Utrecht, Utrecht, The Netherlands; De Kinderkliniek, Almere, The Netherlands

**Keywords:** Pulmonary physiology, Ventilation, Breathing pattern, Children

## Abstract

Data regarding the ventilatory response to exercise in adolescents with mild-to-moderate cystic fibrosis (CF) are equivocal. This study aimed to describe the ventilatory response during a progressive cardiopulmonary exercise test (CPET) up to maximal exertion, as well as to assess the adequacy of the ventilatory response for carbon dioxide (CO_2_) exhalation. Twenty-two adolescents with CF (12 boys and 10 girls; mean ± SD age: 14.3 ± 1.3 years; FEV_1_: 78.6 ± 17.3% of predicted) performed a maximal CPET. For each patient, data of a sex- and age matched healthy control was included (12 boys and 10 girls; mean ± SD age: 14.3 ± 1.4 years). At different relative exercise intensities of 25%, 50%, 75%, and 100% of peak oxygen uptake (VO_2peak_), breathing pattern, estimated ventilatory dead space ventilation (VD/VT ratio), minute ventilation (VE) to CO_2_ production relationship (VE/VCO_2_-slope), partial end-tidal CO_2_ tension (P_ET_CO_2_), and the VE to the work rate (VE/WR) ratio were examined. VO_2peak_ was significantly reduced in CF patients (P = 0.01). We found no differences in breathing pattern between both groups, except for a significantly higher VE at rest and a trend towards a lower VE at peak exercise in patients with CF. Significantly higher values were found for the estimated VD/VT ratio throughout the CPET in CF patients (P < 0.01). VE/VCO_2_-slope and P_ET_CO_2_ values differed not between the two groups throughout the CPET. VE/WR ratio values were significantly higher in CF during the entire range of the CPET (P < 0.01). This study found an exaggerated ventilatory response (high VE/WR ratio values), which was adequate for CO_2_ exhalation (normal VE/VCO_2_-slope and P_ET_CO_2_ values) during progressive exercise up to maximal exhaustion in CF patients with mild-to-moderate airway obstruction.

## Background

Peak oxygen uptake (VO_2peak_) is reported to be limited in patients with cystic fibrosis (CF) (Bongers et al. 2012; Hjeltnes et al. 1984; Keochkerian et al. 2008; Shah et al. 1998; Wideman et al. 2009). This reduction seems to have a multifactorial cause (Selvadurai et al. 2002). Respiratory, cardiovascular, as well as peripheral muscle function are reported as potential exercise limiting mechanisms (Almajed and Lands 2012). In patients with mild to moderate pulmonary disease, non-pulmonary factors, such as low muscle mass, impaired skeletal muscle function and centrally mediated oxygen delivery, seem to predominate in limiting exercise capacity (Regnis et al. 1996; Moorcroft et al. 2005; Saynor et al. 2014). In more severe patients with CF (forced expiratory volume in one second (FEV_1_) <60% of predicted), ventilatory constraints and impaired gas exchange become more important determinants.

Due to continuous airflow obstruction, as reflected by a decreased FEV_1_ and dynamic hyperinflation, adolescents with CF have been described to develop a rapid shallow breathing pattern at rest (Hart et al. 2002) and during exercise (Keochkerian et al. 2005). This can be accompanied with a decreased ventilatory capacity and concomitant reduced VO_2peak_ (Keochkerian et al. 2008). Children and adolescents with CF with static hyperinflation at rest (residual volume to total lung capacity ratio (RV/TLC) >30%) seem to be more prone to a ventilatory limitation during exercise, which appears to be associated with decreased exercise performance (Sovtic et al. 2013; Werkman et al. 2010).

A recent study found that exercise limitation in adult patients with CF is multifactorial and that it was dominantly correlated with FEV_1_ and nutritional and inflammatory status, but also with the magnitude of the overall ventilatory response during exercise (Pastré et al. 2014). In patients with severe airway obstruction (FEV_1_ < 50% of predicted), multivariate analysis revealed the FEV_1_ to be a significant independent predictor of exercise capacity, whereas the ratio between minute ventilation and carbon dioxide exhalation (VE/VCO_2_ ratio) at peak exercise was the major determinant of exercise limitation in patients with mild-to-moderate disease (FEV_1_ > 50% of predicted) (Pastré et al. 2014). However, knowledge about the ventilatory response to exercise in adolescent patients with mild to moderate CF is ambiguous and inconclusive. Several studies in mild-to-moderate adolescents with CF describe an exaggerated ventilatory response with a rapid shallow breathing pattern at rest (Hart et al. 2002) and during exercise (Keochkerian et al. 2008). On the contrary, a recent study in adolescents with mild CF did not find any evidence for a different ventilatory response and/or rapid shallow breathing pattern during exercise (Borel et al. 2014). As the two studies demonstrating exaggerated ventilatory responses were performed in adolescents with CF with lower FEV_1_ values, this suggests that the ventilatory response to exercise is at least partially affected by the degree of airway obstruction.

Moreover, questions can be raised whether the adopted rapid shallow breathing pattern is beneficial as higher breathing frequencies seem to increase ventilatory dead space ventilation (VD/VT ratio), as has been reported in patients with CF waiting for lung transplantation (Thin et al. 2004). However, adding additional dead space volume during exercise in patients with mild CF lung disease had no influence on VO_2peak_ and the duration of the exercise test, and it even increased ventilation which was attributed to an increased tidal volume with no change in respiratory rate (Dodd et al. 2006). The increased dead space volume was accompanied by higher VE/VCO_2_ ratios during exercise (Dodd et al. 2006). This finding in patients with mild CF suggests that the ventilatory response during exercise in adolescents with CF might differ with healthy controls and that this might alter during the course of the disease. Unfortunately the recent study of Borel et al. (2014), which focussed on ventilation during the entire range of exercise, only mentioned mechanical constraints influencing ventilation and did not discuss metabolic issues related to ventilation. Moreover, they included small and unequal groups of only prepubertal children.

As lung function decreases over time in most patients with CF, exercise capacity eventually becomes limited by the lungs reaching their mechanical limits to expand (approximately at FEV_1_ values ≤60%pred) (Almajed and Lands 2012). Insight in the breathing pattern during progressive exercise in adolescents with CF in a broad spectrum of lung function deterioration, as well as the adequacy of this ventilatory response for carbon dioxide (CO_2_) exhalation, is clinically relevant for future therapeutic interventions. Therefore, the aim of the current study was 1) to describe the ventilatory response during a progressive cardiopulmonary exercise test (CPET) up to maximal exertion in adolescents with mild-to-moderate CF and 2) to assess the adequacy of the ventilatory response for CO_2_ exhalation (determined from partial end-tidal carbon dioxide tension (P_ET_CO_2_)) throughout the CPET in mild-to-moderate patients with CF. Our hypothesis is that adolescents with CF develop an obstructive breathing pattern, combined with a relatively large VD/VT ratio, which limit CO_2_ wash out.

## Methods

### Participants

Exercise data of twenty-two adolescents (12 boys and 10 girls from 12 to 17 years of age, mean ± SD age: 14.3 ± 1.3 years) with mild-to-moderate CF were randomly selected from the exercise database from the CF Center at the University Medical Center Utrecht. The database contained anonymous patient data of anthropometry, lung function and exercise capacity which was measured as part of usual care at the routine annual check-up. Therefore, all patients were free from acute pulmonary or gastrointestinal exacerbation at the time of testing. Testing procedures used in this study met the assumptions for standard of practice for the routine care of patients with CF. For each patient with CF, an age, sex and anthropometrically matched healthy control who performed a maximal CPET in our hospital retrospectively was selected (untrained and normal physical activity level). All participants and their guardians provided approval for inclusion of the data in research studies. After evaluation, the medical ethical committee of the University Medical Center Utrecht determined that inclusion of the data conformed to the regulations of the Dutch CF Registration and that inclusion of the data in this study met the ethical polices of the University Medical Center Utrecht, as well as the regulations of the Dutch government.

### Anthropometric measures

Body mass and body height were determined using an electronic scale (Seca, Hamburg, Germany) and a stadiometer (Ulmer Stadiometer, Ulm, Germany) respectively. Body mass index (BMI) was calculated as the body mass in kilograms divided by the square of the body height in meters. Standard deviation (SD) scores were calculated for body height for age, body mass for age, and BMI for age using Dutch normative values (Fredriks et al. 2000). The equation of Haycock et al. (1978), validated in infants, children, and adults, was used to obtain the participants’ body surface area (BSA).

### Spirometry and plethysmography

In the patients with CF, spirometry and plethysmography were performed by qualified lung function analysts of the CF Center at the University Medical Center Utrecht. Since the healthy controls were not known with any disease, spirometry and plethysmography was not performed in this group. In order to prevent the potential influence of bronchial reactivity during exercise, spirometry and plethysmography were performed after bronchodilation with salbutamol (800 μg). FEV_1_ was obtained from flow volume curves (Masterscreen, Jaeger, Würzburg, Germany). Residual volume (RV) and total lung volume (TLC) were determined in a body plethysmograph (Master Lab system, Jaeger, Würzburg, Germany). The RV was expressed as a percentage of TLC (RV/TLC ratio) as well. In order to improve comparative possibilities with the reports of other studies in CF, we used the commonly used reference values of Zapletal et al. (1987) to express lung function values as percentage of predicted values.

### Cardiopulmonary exercise test

All participants completed a maximal CPET using an electronically braked cycle ergometer (Jaeger Physis, Carefusion, Houten, The Netherlands) after bronchodilation with salbutamol. After three minutes of rest measurements, cycling started at a workload of 0 W. Then, the work rate (WR) was linearly incremented with a 15 W∙min^−1^ ramp protocol (Godfrey 1974) until the patient stopped due to volitional exhaustion, despite strong verbal encouragement. The test effort was considered maximal when the participant showed objective (heart rate (HR) at peak exercise (HR_peak_) >180 beats · min^−1^ and/or a respiratory exchange ratio (RER) at peak exercise (RER_peak_) >1.0) (Armstrong and Welsman 2008) and subjective (unsteady biking, sweating, facial flushing, and clear unwillingness to continue despite encouragement) signs of maximal effort.

Throughout the CPET, participants breathed through a facemask (dead space volume 63 or 72 mL, dependent on size) (Hans Rudolph Inc, Kansas City, MO) that was connected to a calibrated metabolic cart (Oxycon Pro, Carefusion, Houten, the Netherlands). Gas analyzers were calibrated using gases of known concentration, whereas the flow meter was calibrated using a three-liter syringe (Hans Rudolph Inc, Kansas City, MO). Expired gas passed through a flow meter and a gas analyzer connected to a computer, which calculated breath-by-breath minute ventilation (VE), oxygen uptake (VO_2_), VCO_2_ and RER from conventional equations. Output from the flow meter and gas analyzers were averaged over ten-second intervals and stored for further use. Relative VO_2peak_ (VO_2peak_/kg) was calculated by dividing VO_2peak_ by body mass. HR was monitored continuously by a three-lead electrocardiogram (Hewlett-Packard, Amstelveen, Netherlands) and transcutaneous O_2_ saturation at the index finger was measured by pulse oximetry (Nellcor 200 E, Nellcor, Breda, the Netherlands). Peak exercise parameters were defined as the highest values achieved within the last 30 seconds prior to exhaustion.

### Data analysis

The ventilatory threshold was defined as the point at which the ventilatory equivalent for oxygen and the partial end-tidal oxygen tension reached a minimum and thereafter began to rise in a consistent manner, coinciding with an unchanged ventilatory equivalent for carbon dioxide and a peak in the P_ET_CO_2_ course (American Thoracic Society, American College of Chest Physicians 2003; Ohuchi et al. 1996). When this ventilatory equivalents method appeared to provide uncertain results for a participant’s ventilatory threshold (n = 4, 18%, in the CF patients, n = 0 in the healthy controls), the point at which the linear slope of the relation between the VCO_2_ and VO_2_ changed was taken as the ventilatory threshold, according to the V-slope method (Beaver et al. 1986). The ventilatory threshold (VO_2_) was expressed as an absolute value, relative value (VO_2_ normalized for body mass) and as a percentage of the attained VO_2peak_. The estimated VD/VT ratio was calculated by using the P_ET_CO_2_. The graphical presentation of VE as a function of VCO_2_ during the progressive CPET was used to determine the point at which VE increased out of proportion to VCO_2_, the respiratory compensation point.

Next to resting values, tidal volume, breathing frequency, VE, VD/VT ratio, and P_ET_CO_2_ were determined as an average of 30 seconds at different exercise intensities of 25%, 50%, 75% and 100% of VO_2peak_. Both VE and tidal volume were adjusted for body mass as well. To allow for fair comparison, resting VO_2_ was subtracted from VO_2peak_. Subsequently, 25%, 50%, 75% and 100% of this delta VO_2_ was calculated and summed with the resting VO_2_ for each participant (CF patients and healthy controls). Furthermore, we calculated the VE to VO_2_ relationship (VE/VO_2_-slope) and the VE to VCO_2_ relationship (VE/VCO_2_-slope) at the same different exercise intensities by linear regression of the exercise data up to 25%, 50%, 75%, and 100% of VO_2peak_ using the least squares approach. We examined the participants’ response of the VE to the WR (VE/WR ratio) at similar intensities. Data from the first minute of exercise were excluded since the breathing pattern during the first minute of exercise frequently appears to be unstable.

### Statistical analysis

The Statistical Package for the Social Sciences (SPSS, version 15.0; SPSS Inc., Chicago, IL) was used for the data-analysis. Shapiro-Wilk tests for normality were performed in order to evaluate the data distribution of each variable. Differences between adolescents with CF and their healthy counterparts in anthropometry and in exercise data at different exercise intensities were examined with Mann–Whitney U tests. Data are presented as mean values ± SD. A P-value <0.05 was considered as statistically significant.

## Results

Anthropometric data for the patients with CF and the healthy controls are presented in Table 1, with no significant anthropometric between-group differences. Lung function characteristics of the adolescents with CF are also shown in Table 1. Patients had mild-to-moderate airway obstruction (mean FEV_1_ expressed as a percentage of predicted of 79 ± 17%) and a mild-to-moderate degree of static hyperinflation (mean RV/TLC ratio of 34 ± 9%). More specifically, 14 patients (5 boys and 9 girls) had an absolute RV/TLC ratio greater than 30%, which suggests static hyperinflation (Eid et al. 2000).

**Table 1:** **Participant characteristics**

	Healthy (n = 22)	CF (n = 22)	*P*-value
Sex (boy/girl)	12/10	12/10	*NA*
Age (years)	14.3 ± 1.4	14.3 ± 1.3	0.99
CF mutation class (I/II/IV/unknown)^a^	*NA*	9/27/1/6	*NA*
PA colonization (never/free of infection/intermittent/chronic)^b^	*NA*	6/1/6/9	*NA*
Body height (m)	1.67 ± 0.10	1.65 ± 0.09	0.61
Body height for age SDS^c^	0.17 ± 0.86	−0.18 ± 0.99	0.24
Body mass (kg)	53.9 ± 12.2	50.2 ± 7.2	0.31
Body mass for age SDS^c^	0.02 ± 0.83	−0.37 ± 0.64	0.13
BMI (kg · m^−2^)	19.1 ± 2.6	18.5 ± 2.0	0.51
BMI for age SDS^c^	−0.09 ± 0.80	−0.34 ± 0.85	0.37
BSA (m^2^)	1.57 ± 0.22	1.54 ± 0.14	0.72
FEV_1_ (L)	*NA*	2.52 ± 0.67	*NA*
FEV_1_ (%pred)^d^	*NA*	78.6 ± 17.3	*NA*
RV/TLC (%)	*NA*	33.5 ± 9.3	*NA*
VO_2peak_/kg (mL · kg^−1^ · min^−1^)	49.1 ± 7.2	42.4 ± 8.7	<0.01**

Values are presented as mean ± SD.

*Abbreviations*: *BMI* body mass index, *BSA* body surface area, *CF* cystic fibrosis, *CFTR* cystic fibrosis transmembrane conductance regulator, *FEV*_*1*_ forced expiratory volume in one second, *NA* not applicable, *NS* not statistically significant, *RV/TLC* residual volume to total lung capacity ratio, *SDS* standard deviation score.

**P < 0.01.

^a^Based on the classification of CFTR alleles used by Green et al. (2010).

^b^Based on the criteria of Lee et al. (2003).

^c^Reference values of Fredriks et al. (2000).

^d^Reference values of Zapletal et al. (1987).

All participants performed a maximal effort during the CPET (mean test duration of 623 ± 139 and 659 ± 194 seconds for the CF patients and healthy controls respectively), and the results are presented in Table 2. Compared with the healthy controls, adolescents with CF attained significantly lower values for HR_peak_, peak WR (WR_peak_), WR_peak_/kg, VO_2peak_, VO_2peak_/kg, VO_2peak_/m^2^ (normalized for BSA), peak VCO_2_ (VCO_2peak_), VCO_2peak_/kg, and peak VE (VE_peak_) normalized for body mass (VE_peak_/kg), whereas they attained significantly higher values for the estimated VD/VT ratio at peak exercise, the VE/VO_2_-slope up to the ventilatory threshold, P_ET_CO_2_ at rest, and the VE/WR ratio at peak exercise.

**Table 2:** **Exercise performance in the healthy adolescents and adolescents with CF**

	Healthy (n = 22)	CF (n = 22)	*P*-value
**Maximal values**			
HR_peak_ (beats · min^−1^)	192 ± 7	186 ± 8	0.02*
RER_peak_	1.15 ± 0.08	1.19 ± 0.09	0.16
WR_peak_ (W)	222 ± 62	164 ± 32	<0.01**
WR_peak_/kg (W · kg^−1^)	4.2 ± 0.6	3.3 ± 0.5	<0.001***
VO_2peak_ (mL · min^−1^)	2638 ± 685	2126 ± 516	0.01*
VO_2peak_/kg (mL · kg^−1^ · min^−1^)	49.1 ± 7.2	42.4 ± 8.7	<0.01**
VO_2peak_/BSA (mL · m^2^ · min^−1^)	1667 ± 259	1378 ± 274	<0.01**
VCO_2peak_ (mL · min^−1^)	2963 ± 871	2409 ± 540	0.03*
VCO_2peak_/kg (mL · kg^−1^ · min^−1^)	54.9 ± 8.9	48.1 ± 9.7	0.02*
VE @ VO_2peak_ (L · min^−1^)	89.5 ± 27.2	75.8 ± 18.8	0.08
VE/kg @ VO_2peak_ (L · kg^−1^ · min^−1^)	1.7 ± 0.3	1.5 ± 0.3	0.04*
Absolute tidal volume @ VO_2peak_ (mL)	1736 ± 339	1616 ± 373	0.32
Relative tidal volume @ VO_2peak_ (mL · kg^−1^)	32.7 ± 4.8	32.2 ± 6.4	0.98
Breathing frequency @ VO_2peak_ (breaths · min^−1^)	51 ± 9	48 ± 9	0.25
Estimated VD/VT ratio @ VO_2peak_ (%)	17 ± 2	21 ± 4	<0.001***
P_ET_CO_2_ @ VO_2peak_ (mmHg)	36.3 ± 3.2	38.2 ± 3.7	0.10
VE/WR ratio @ VO_2peak_ (mL · min^−1^ · W^−1^)	399 ± 57	476 ± 71	<0.001***
**Submaximal values**			
Absolute ventilatory threshold (mL · min^−1^)	1439 ± 381	1216 ± 253	0.07
Relative ventilatory threshold (mL · kg^−1^ · min^−1^)	27.1 ± 6.1	24.4 ± 5.1	0.13
Ventilatory threshold (%VO_2peak_)	55 ± 9	58 ± 9	0.32
P_ET_CO_2_ @ rest (mmHg)	31.8 ± 2.4	33.5 ± 3.2	0.03*
P_ET_CO_2_ @ the ventilatory threshold (mmHg)	39.3 ± 3.6	40.0 ± 2.6	0.50
VE/VO_2_-slope up to the ventilatory threshold	20.5 ± 3.2	23.7 ± 5.1	0.02*
VE/VCO_2_-slope up to the respiratory compensation point	26.6 ± 2.8	27.1 ± 2.9	0.60

Values are presented as mean ± SD.

*Abbreviations*: *BSA* body surface area, *CF* cystic fibrosis, *HR*_*peak*_ peak heart rate, *NS* not statistically significant, *P*_*ET*_ *CO*_*2*_ partial end-tidal carbon dioxide tension, *RER*_*peak*_ peak respiratory exchange ratio, *VCO*_*2peak*_ peak carbon dioxide output, *VCO*_*2peak*_ */kg* peak carbon dioxide output normalized for body mass, *VD/VT* ratio physiological dead space ventilation, *VE* minute ventilation, *VE/kg* minute ventilation normalized for body mass, *VE/VCO*_*2*_ *-slope* slope of the relationship between minute ventilation and carbon dioxide output, *VE/VO*_*2*_ *-slope* slope of the relationship between minute ventilation and oxygen uptake, *VE/WR* minute ventilation to work rate ratio, *VO*_*2peak*_ peak oxygen uptake, *VO*_*2peak*_ */kg* peak oxygen uptake normalized for body mass, *WR*_*peak*_ peak work rate, *WR*_*peak*_ */kg* peak work rate normalized for body mass.

*P < 0.05; **P < 0.01; ***P < 0.001.

**Figure 1:**
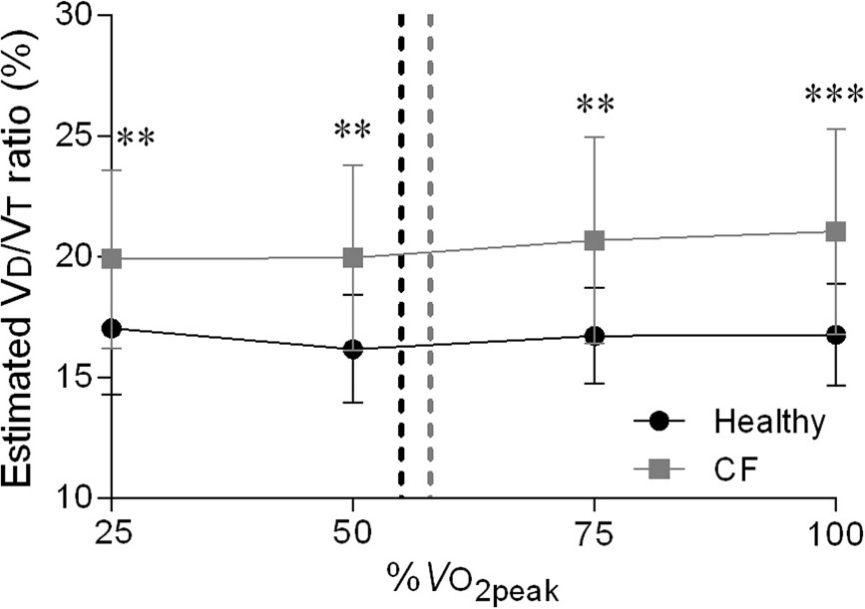
**Changes in the estimated VD/VT ratio during exercise at similar percentages of VO**_**2peak**_ **in the healthy adolescents and the adolescents with CF.** Dashed lines correspond to the ventilatory threshold in both groups. Abbreviations: CF = cystic fibrosis; VD/VT ratio = physiological dead space ventilation; VO_2peak_ = peak oxygen uptake. **P < 0.01; ***P < 0.001.

Breathing pattern components, tidal volume and breathing frequency, adopted during exercise did not differ significantly between patients with CF and healthy adolescents, except for a significantly higher breathing frequency at rest (19 ± 3 versus 22 ± 5 breaths · min^−1^; P = 0.02) and a trend for lower absolute tidal volume values at or near maximal exercise in patients with CF (1.74 ± 0.34 versus 1.62 ± 0.37 L; P = 0.32). Consequently, VE at rest was significantly higher (11.5 ± 2.1 versus 13.8 ± 3.4 L · min^−1^; P = 0.02), whereas VE_peak_ (75.8 ± 18.8 versus 89.5 ± 27.2 L^.^min^−1^; P = 0.08) tended to be lower in patients with CF. VE normalized for body mass (VE/kg) was significantly higher at rest (0.2 ± 0.05 versus 0.3 ± 0.06 L · kg^−1^ · min^−1^; P < 0.01) and at 25% of VO_2peak_ (0.4 ± 0.07 versus 0.5 ± 0.08 L · kg^−1^ · min^−1^; P = 0.03) in the patients with CF. Values for breathing frequency divided by the tidal volume (rapid shallow breathing index) tended to be higher in patients with CF at rest (32 ± 13 versus 38 ± 13; P = 0.14) and at 25% of VO_2peak_ (27 ± 10 versus 32 ± 15; P = 0.31). Furthermore, estimated VD/VT ratio values were significantly higher during all exercise intensities in patients with CF (see Figure 1, P < 0.01, P < 0.01, P < 0.01, and P < 0.001 at 25%, 50%, 75%, and 100% of VO_2peak_ respectively).

**Figure 2:**
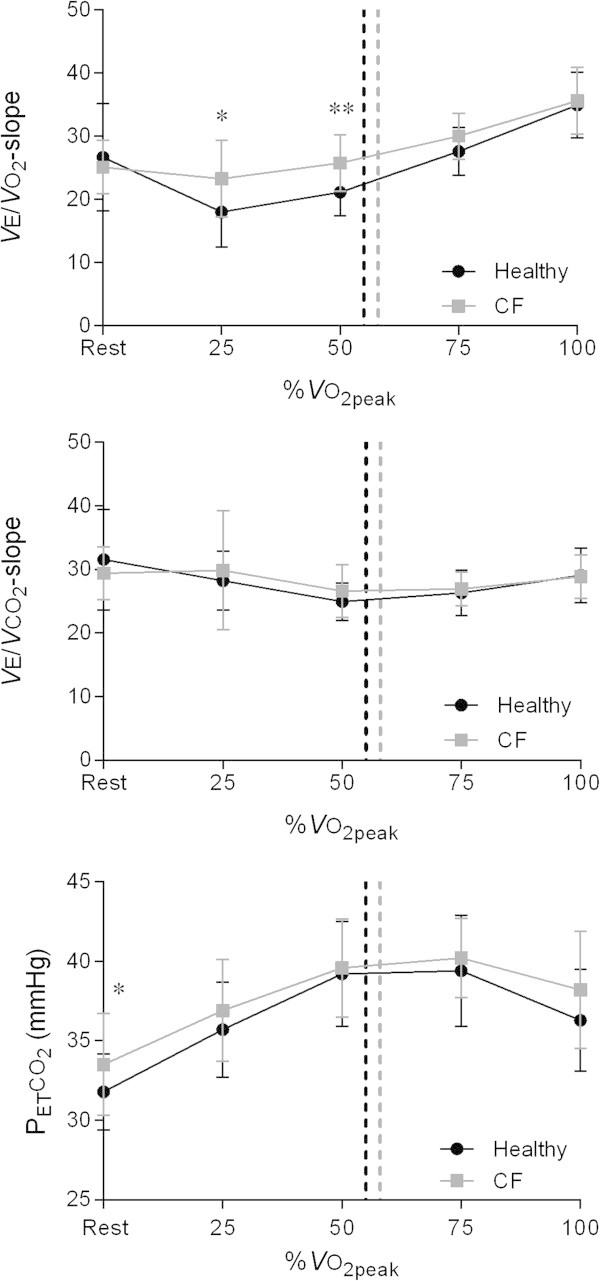
**Changes in VE/** **VO**_**2**_-**slope,** **VE/** **VCO**_**2**_ **-slope,** **and P**_**ET**_ **CO**_**2**_ **during exercise at similar percentages of VO**_**2peak**_ **in the healthy adolescents and the adolescents with CF.** Dashed lines represent the ventilatory threshold in both groups. Abbreviations: CF = cystic fibrosis; P_ET_CO_2_ = partial end-tidal carbon dioxide tension; VE/VCO_2_-slope = slope of the relationship between minute ventilation and carbon dioxide output; VE/VO_2_-slope = slope of the relationship between minute ventilation and oxygen uptake; VO_2peak_ = peak oxygen uptake. *P < 0.05; **P < 0.01.

Figure 2 shows significantly higher VE/VO_2_-slope values at 25% (P = 0.01) and 50% (P < 0.01) of VO_2peak_ in patients with CF, whereas VE/VCO_2_-slope values differed not significantly between the two groups throughout the entire range of the CPET. Furthermore, Figure 2 demonstrates that, except for resting values (P = 0.03), P_ET_CO_2_ values differed not significantly between the two groups during the CPET.

**Figure 3:**
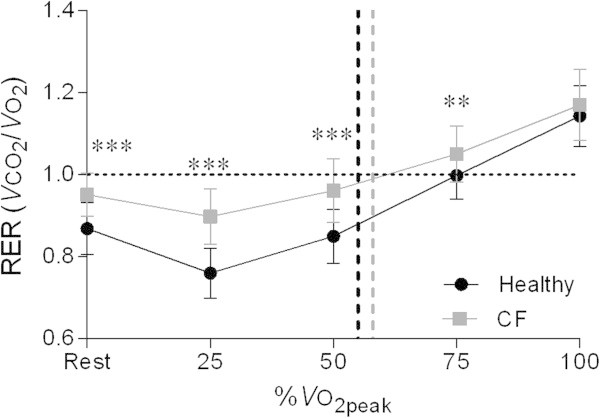
**RER during exercise at similar percentages of VO**_**2peak**_ **in healthy adolescents and patients with CF.** Vertical dashed lines correspond to the ventilatory threshold in both groups. Abbreviations: CF = cystic fibrosis; RER = respiratory exchange ratio; VO_2peak_ = peak oxygen uptake. **P < 0.01; ***P < 0.001.

**Figure 4:**
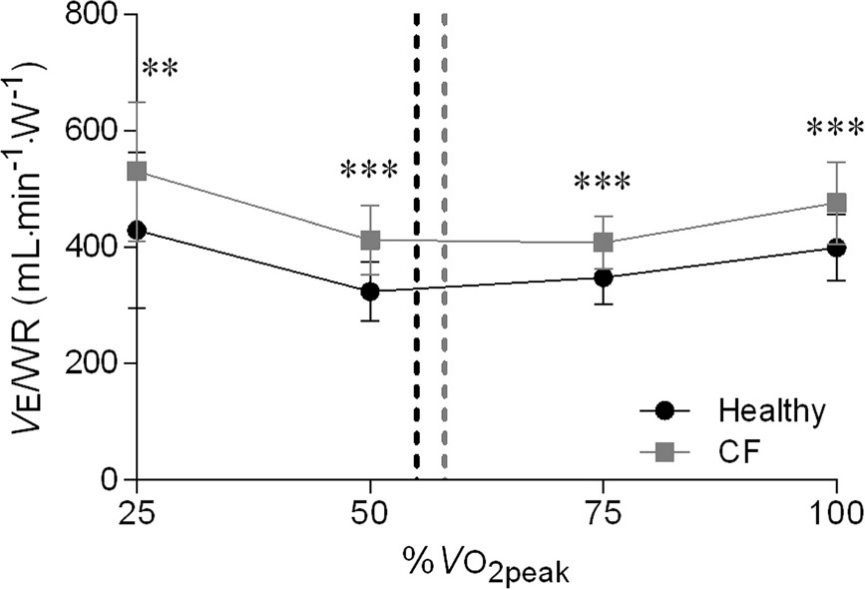
**Changes in the VE** **/** **WR ratio during exercise at similar percentages of VO**_**2peak**_ **in healthy adolescents and patients with CF.** Dashed lines correspond to the ventilatory threshold in both groups. Abbreviations: CF = cystic fibrosis; VE/WR = minute ventilation to work rate ratio; VO_2peak_ = peak oxygen uptake. **P < 0.01; ***P < 0.001.

Figure 3 represents the RER at rest and throughout progressive exercise in order to elucidate the higher VE/VO_2_ ratios during sub-maximal exercise. At rest and during sub-maximal exercise, patients with CF attained significantly higher RER values (P < 0.001, P < 0.001, P < 0.001, P < 0.01, and P = 0.26 at rest, 25%, 50%, 75%, and 100% of VO_2peak_ respectively). The VE/WR ratio depicted in Figure 4 was significantly higher in CF during the entire range of the CPET (P < 0.01, P < 0.001, P < 0.001, and P < 0.001 at 25%, 50%, 75%, and 100% of VO_2peak_ respectively).

## Discussion

The present study aimed to 1) describe the ventilatory response during a progressive CPET and 2) assess the adequacy of the ventilatory response for CO_2_ exhalation during exercise in mild-moderate adolescents with CF. First, we found an exaggerated ventilatory response during exercise. Second, this ventilatory response to exercise seems to be adequate for CO_2_ exhalation in patients with mild-to-moderate CF. The latter is illustrated by a similar course of the VE/VCO_2_-slope throughout exercise, higher RER values during sub-maximal exercise, and the ability to maintain P_ET_CO_2_ values within normal limits throughout the entire range of the CPET.

The basic physiological factors that determine and modify the ventilatory response to exercise are: 1) CO_2_ output of the exercising muscles, 2) the arterial CO_2_ set-point, 3) the VD/VT ratio, and 4) the change in the arterial pressure of CO_2_ (PaCO_2_) during exercise (Wasserman et al. 1996). Several of these concepts are addressed to in this study. First, muscular CO_2_ output during (sub-)maximal exercise seems to be increased in patients with CF (Bongers et al. 2012; Hebestreit et al. 2005; Nguyen et al. 2014), which is illustrated in the current study by a higher RER. The higher RER in patients with CF is suggested to reflect a higher reliance on glucose oxidation to meet energy demands during exercise (Hebestreit et al. 2005; Nguyen et al. 2014). Altered substrate utilization in CF (de Meer et al. 1995; Moser et al. 2000; Selvadurai et al. 2003) might explain the increased RER at rest and for the lower exercise intensities. In addition, slowed VO_2_ kinetics during exercise and recovery in patients with CF might also be a possible explanation for an increased RER during sub-maximal exercise (Kusenbach et al. 1999; Massin et al. 2000; Pouliou et al. 2001; Hebestreit et al. 2005; Stevens et al. 2009; Saynor et al. 2014). This results in a greater dependency on glycolytic energy systems at lower exercise intensities resulting in an increased VCO_2_. Higher aerobic or anaerobic glycolytic energy expenditure during exercise increases ventilation as supported by a study in patients with familial mitochondrial myopathy (Heinicke et al. 2011). The current study showed an exaggerated VE relative to metabolic rate, indicated by high VE/VO_2_-slope values and VE/WR ratios, as well as an elevated RER, with no apparent signs of pulmonary insufficiency.

Second, the VD/VT was significantly higher at all exercise intensities in patients with CF, which seems to be exaggerated with disease progression (post-hoc analysis, data not shown). High VD/VT values during exercise have previously been reported in patients with CF (Cerny et al. 1982; Coates et al. 1988; Thin et al. 2004; Wilkens et al. 2010). This, in combination with the trend of higher P_ET_CO_2_ in patients with CF, indicates an abnormal alveolar dead space ventilation (Wilkens et al. 2010), which might explain the relatively high VE values at rest and during sub-maximal exercise in patients with CF. Unfortunately, the exaggerated ventilatory response we found and the shift from oxidative to glycolytic energy metabolism, as shown by the attenuation of the RER slope at exercise intensities above 50% of VO_2peak_ when compared to healthy controls, seems not to be able to totally compensate the limited exercise capacity in patients with CF (significantly reduced VO_2peak_/kg and WR_peak_/kg). Our results are in agreement with the study of Borel et al. (2014) who found no effect of mild CF on breathing pattern and breathing strategy during an incremental CPET. Although breathing pattern and breathing strategy of adolescents with CF were comparable with healthy controls, most studies still report a reduced exercise capacity in adolescents with CF (Almajed and Lands 2012; Rand and Prasad 2012; Saynor et al. 2014). Saynor et al. (2014) recently suggested that centrally mediated oxygen delivery might be the principally limiting the aerobic function of pediatric CF patients with mild-to-moderate airway obstruction during ramp incremental cycling exercise. However, based on the results of the study of Keochkerian et al. (2008) on the ventilatory response in children with CF, one would still expect that CF could alter the ventilatory response during a progressive CPET. The current study demonstrated no significant effect of CF on the ventilatory response for CO_2_ exhalation (VE/VCO_2_-slope and P_ET_CO_2_) to exercise in patients with CF with mild-to-moderate airway obstruction.

As described above, we found higher values for RER, VE/WR, and VE/VO_2_-slope values during sub-maximal exercise. These results suggest a higher ventilatory demand rather than a higher ventilatory response during sub-maximal exercise in patients with mild CF. An explanation for the absence of an impact of CF on the ventilatory response to exercise is the mild-to-moderate severity of the lung disease in our population. Keochkerian et al. (2008) reported that the more severe the airway obstruction, the more rapid and shallow the breathing pattern.

The current study has some limitations. Firstly, by categorizing exercise intensity by %VO_2peak_, there is no standardization of exercise intensity relative to the ventilatory response for each participant. However, standardizing for exercise intensity relative to VO_2_ (%VO_2peak_) was also done by other studies (Borel et al. 2014; Keochkerian et al. 2008). Since the ventilatory threshold occurred at a similar percentage of VO_2peak_ in the pediatric CF patients and the healthy controls (58 ± 9% versus 55 ± 9%; P = 0.32), we believe that we did compare groups within the same physiological exercise intensity domain. Secondly, the sample size was relatively small and included mainly patients with CF with mild to moderate airflow obstruction. For this reason, these findings cannot be generalized to patients with severe airflow obstruction. Nevertheless, the current study sample is representative for the CF population in a tertiary CF Center. Thirdly, the estimated VD/VT ratio cannot be accurately predicted from the P_ET_CO_2_ in patients with an increased VD/VT ratio due to lung disease (Wasserman et al. 2005), so caution must be taken with the interpretation of these results. Fourth, unfortunately we were not able to correct VO_2peak_ for fat free mass as this variable was not routineously measured in patients with CF in the CF Center at the University Medical Center Utrecht. Finally, the used criteria for a maximal effort are subject to debate, especially in patients with CF. It has previously been demonstrated that traditional testing protocols and verification criteria significantly underestimate VO_2peak_ in both healthy (Barker et al. 2011) and children with CF (Saynor et al. 2013). We did not verify the attainment of a true VO_2peak_ with a supramaximal exercise testing procedure.

### Implications and future research

As a higher ventilatory demand seems to be present during submaximal exercise in mild-moderate patients with CF, a small decline in ventilatory capacity might hamper the precarious balance between ventilation and homeostasis with further disease progression in patients with CF. The main findings presented in this study highlight the importance for the clinician to aim for attenuation of lung function decline even in patients with CF with a relatively preserved lung function (“normal” FEV_1_). For future research it would be interesting to evaluate the latter hypothesis in patients with CF with more severe airway obstruction. Moreover, the differences we found during the course of sub-maximal exercise highlight the importance to evaluate the submaximal exercise response as well when interpreting a CPET, and not just focus on peak exercise parameters.

## Conclusions

The current study found an exaggerated, but adequate ventilatory response to exercise for CO_2_ exhalation in patients with CF with mild-to-moderate airway obstruction. The higher RER, VE/WR ratios, and VE/VO_2_-slope values during sub-maximal exercise point towards a higher ventilatory demand during sub-maximal exercise in patients with CF and mild-to-moderate lung disease.

## Abbreviations

BMI: Body mass index
BSA: Body surface area
CF: Cystic fibrosis
CO_2_: Carbon dioxide
CPET: Cardiopulmonary exercise test
FEV_1_: Forced expiratory volume in one second
HR: Heart rate
HR_peak_: peak heart rate
P_ET_CO_2_: Partial end-tidal carbon dioxide tension
RER: Respiratory exchange ratio
RER_peak_: Respiratory exchange ratio at peak exercise
RV: Residual volume
RV/TLC ratio: Residual volume to total lung capacity ratio
SD: Standard deviation
TLC: Total lung capacity
VCO_2_: Carbon dioxide production
VCO_2peak_: Peak carbon dioxide production
VD/VT ratio: Ventilatory dead space ventilation ratio
VE: minute ventilation
VE_peak_: peak minute ventilation
VE/VO_2_-slope: minute ventilation to oxygen uptake slope
VE/VCO_2_ ratio: minute ventilation to carbon dioxide production ratio
VE/VCO_2_-slope: minute ventilation to carbon dioxide production slope
VE/WR: minute ventilation to work rate ratio
VO_2_: Oxygen uptake
VO_2peak_: peak oxygen uptake
WR: Work rate
WR: peak work rate.
